# Linear Motion Coverage as a Determinant of Transparent Liquid Perception

**DOI:** 10.1177/2041669518813375

**Published:** 2018-12-08

**Authors:** Takahiro Kawabe

**Affiliations:** NTT Communication Science Laboratories, Atsugi, Japan

**Keywords:** dynamic image deformations, linear motion, surface perception, transparent liquid

## Abstract

When a transparent liquid flows, the background image behind the flow dynamically deforms due to light refraction. The dynamic deformations of a background image (dynamic image deformations) are one of the visual features used by the visual system to infer the existence of a transparent liquid flow. Although previous studies have discussed the role of the narrow band components of the spatiotemporal deformation frequency, it was still unclear whether motion signals, one of the constituents of dynamic image deformations, were the determinant of the perception of a transparent liquid. Manipulating the flow speed of image deformation, which is a critical parameter for changing motion signals in dynamic image deformations, we asked observers to judge whether a transparent liquid was included in the clips or not. We found that the proportions of reporting that they saw a transparent liquid increased with the flow speed of image deformations. Analyzing motion signals of the stimulus clips, we found that the faster the flow of image deformations the fewer linear motion signals were contained. The results indicate that the perception of a transparent liquid arises when the dynamic image deformations contain fewer linear motion signals.

## Introduction

The visual system takes various approaches to infer the presence of materials or stuff (Adelson, 2001; Adelson & Bergen, 1991). For example, in order to infer the optical properties of materials, such as specular reflection, the visual system adopts various approaches including inverse-optics (Marr, 1982), heuristics on the basis of image statistics (Motoyoshi, Nishida, Sharan, & Adelson, 2007; Nishida & Shinya, 1998), and the assessment of image feature appearances (Fleming, 2014; Marlow, Kim, & Anderson, 2012). The combination of some of these approaches may make it possible for the visual system to robustly infer the properties of materials, which could be the basis of the capacity for the effortless recognition of materials in everyday scenarios.

Among the various types of materials, the present study focuses on a transparent liquid. Transparent volumetric materials often cause the image deformation of their backgrounds due to light refraction if the materials have a refractive index of more than 1. When a transparent liquid flows, the image deformation that is produced by the transparent liquid becomes dynamic. We refer to the dynamic nature of image deformations as dynamic image deformations. Recent studies have shown that dynamic image deformations are used by the visual system to judge the state (i.e., solid, liquid, or gas) of a transparent material (Kawabe, 2017; Kawabe & Kogovšek, 2017; Kawabe, Maruya, & Nishida, 2015). For example, Kawabe et al. (2015) investigated which bands of the spatiotemporal deformation frequency led to the impression of a transparent liquid. Kawabe et al. reported that a narrow band of the spatiotemporal deformation frequency played a key role in a liquid being perceived as transparent. A different study (Kawabe & Kogovšek, 2017) sought to identify what feature was critical for differentiating hot air from water, both of which are transparent materials, and found that the magnitude of dynamic image deformations was a vital image cue for human observers to differentiate them. These previous studies have used stimulus clips wherein the entire spatial region of the clip was filled with the dynamic image deformations of a background scene. On the other hand, a more recent study (Kawabe, 2017) used a stimulus clip wherein only the disk-shaped area of the clip contained dynamic image deformations. Thus, the area had a circular contour that was defined by the presence or absence of dynamic image deformations. By using these kinds of stimuli, Kawabe (2017) reported that not only dynamic image deformations within the disk-shaped area but also dynamic changes in contour shape of the area containing dynamic image deformations were important features that determined the perception of the deforming region as either a solid or liquid material.

An important question to be addressed is what types of motion signals in dynamic image deformations cause the perception of a transparent liquid. Previous studies have shown that some attributes of dynamic image deformations are an important cause of transparent liquid perception (Kawabe, 2017; Kawabe & Kogovšek, 2017; Kawabe et al., 2015). However, to date, neither psychophysical nor neuroscience studies have provided evidence to show that the visual system has detectors that are dedicatedly sensitive to dynamic image deformations. Rather, the possibility exists that motion signals that are inherently contained in dynamic image deformations are the determinant of the perception of a transparent liquid. We believe that it is thus necessary to assess how motion signals in dynamic image deformations can explain the perception of a transparent liquid, without simply ascribing it to the attributes of dynamic image deformations.

**Figure 1.**
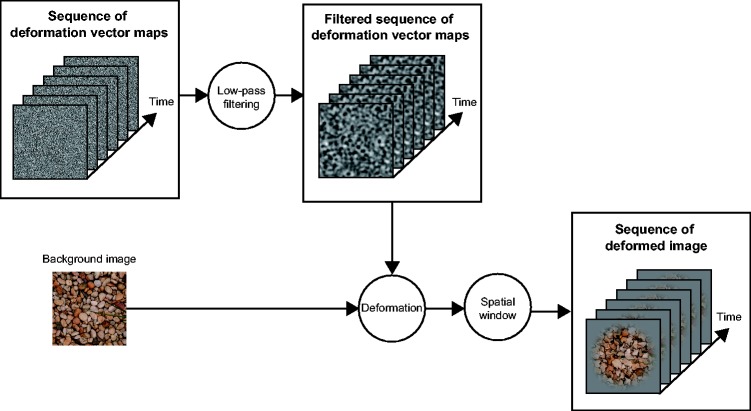
Schematic diagram showing how stimulus images were generated.

The present study investigated how the flow speed of image deformations affected the perception of a transparent liquid. To deform a background image, we used deformation vector maps that store the degree to which each pixel of the image is moved (Figure 1, see Method section for details on how the stimulus images were created). By horizontally shifting the maps and sequentially deforming the background image on the basis of the shifted maps, the flow of image deformations could be created. When the magnitude of the shift was small, that is, the flow speed of image deformations was slow, a particular area in the background image is sequentially deformed by the adjacent parts of the deformation vector maps across frames (Video 1). Because the values in the deformation vector maps gradually change across the adjacent parts, the particular area in the background image undergoes pixel shifts in similar directions. Hence, in this scenario, linear motion signals, that is, motion signals with a consistent motion direction, are likely generated across the frames (see Results and Discussion section for a further explanation of linear motion). On the other hand, when the magnitude of the shift of the deformation map was large, that is, the flow speed of image deformations was high, a particular area in the background image is sequentially deformed by the disjunct parts of the deformation vector maps across frames (Video 2). Because the values in the deformation vector maps are occasionally very different across adjacent parts, a particular area in the background image occasionally undergoes pixel shifts in different directions across frames. Hence, in this scenario, linear motion signals were generated to a lesser degree. In this way, by manipulating the flow speed of image deformations, it was possible to control the coverage of linear motion in the video clip. In the following experiment, we asked our observers to judge whether the stimulus clip contained a transparent liquid or not. We also analyzed the relationship between the proportion of reporting a transparent liquid and the coverage of linear motion in the clip. Based on the results, we discuss the proposition that the perception of a transparent liquid is based on small coverage of linear motion signals in dynamic image deformations.

**Video 1:** (Click to play).

**Video 2:** (Click to play).

## Experiment

### Method

#### Observers

Twelve naive people (seven females and five males) participated in the experiment. Their mean age was 32.3 years with a standard deviation of 9.12. They reported having normal or corrected-to-normal visual acuity. They were recruited from outside the laboratory and received payment for their participation. Ethical approval for this study was obtained from the ethical committee of Nippon Telegraph and Telephone Corporation (Approval number: H28-008 by NTT Communication Science Laboratories Ethical Committee). The experiments were conducted according to the principles that have their origin in the Helsinki Declaration. Written informed consent was obtained from all participants.

#### Apparatus

Stimuli were presented on a 21-in. iMac (Apple Inc., USA) with a resolution of 2048 × 1152 pixels and a refresh rate of 60 Hz. The outputs of the monitor were gamma corrected. The CIE coordinates of the maximum intensity for each of RGB channel were R (*x* = 0.6675, *y* = 0.3265, 37.8 cd/m^2^), G (*x* = 0.2575, *y* = 0.7082, 110.0 cd/m^2^), and B (*x* = 0.1434, *y* = 0.0456, 9.68 cd/m^2^), which were measured using a colorimeter (Bm-5A, Topcon, Japan). The refresh rate of the monitor was 60 Hz. A computer (iMac, Apple Inc., USA) controlled stimulus presentation, and data were collected with PsychoPy v1.83 (Peirce, 2007, 2009).

#### Stimuli

Figure 1 shows how we generated our stimulus clips. Because the procedure used to generate the stimulus clips was complicated, we describe our procedure by explaining each step in more detail later.

*– Preparation of background image.* In the clip, five natural images which were downloaded from the McGill Calibrated Colour Image Database (Olmos & Kingdom, 2004) were used as background images. The size of the background image was 256 × 256 pixels (5.2° × 5.2° in an actual observation).
*– Generation of the sequence of deformation vector map .*The background image was deformed using an image warp technique (Glasbey & Mardia, 1998). The image warp technique uses a deformation vector map by which each pixel in a background image is shifted. In this study, the deformation vector map, whose spatial size was identical to the size of background images, was a two-dimensional matrix wherein each value was drawn from a uniform distribution ranging from 0 to 1. In the present study, we wanted to create a flow of image deformations. Hence, the deformation vector map was horizontally shifted to the right or left. For each clip, the magnitude of the shift was randomly chosen from the following 20 levels: 0.0104°, 0.0109°, 0.0115°, 0.0122°, 0.0130°, 0.0139°, 0.0149°, 0.0160°, 0.0173°, 0.0189°, 0.0208°, 0.0231°, 0.0260°, 0.0297°, 0.03467°, 0.04160°, 0.0520°, 0.0693°, 0.1040°, and 0.2080° of visual angle. The vacant cells in the matrix were filled with the values drawn from the uniform distribution described earlier. Because we wanted the stimulus clip have a duration of 2 s, we repeated this shift 120 times. Consequently, we were able to obtain the sequence of the deformation vector maps.
*– Further modification of the sequence of the deformation vector maps.* Each of the deformation vector maps in the sequence was low-pass filtered with the cutoff frequency of the filter set to 16 cpi (3.08 cycles per deg). The cutoff frequency value was determined in order to simulate the spatial deformation frequency which a real transparent liquid flow was likely to generate (Kawabe et al., 2015). After filtering, the amplitude of the deformation vector maps was modulated so that the vector values ranged between −12 pixels (−0.25°) and 12 pixels (0.25°). Different sequences of the deformation vector maps were used for horizontal and vertical deformation, respectively.
*– Sequential deformations of the background image*. To obtain a stimulus clip, one of the five background images was sequentially deformed by the sequences of the deformation vector maps created in the way described earlier. For example, the *n*th frame of the clip was generated by deforming the background image with the *n*th matrix in the sequence of the deformation vector maps. Because we wanted to create a stimulus clip with a duration of 2 s, *n* was set to 120.
*– Spatial window*. To reduce the visibility of the image deformation artifacts that often arose at the boundary of the background image, we applied a spatial Tukey window to the deformed image.

#### Procedure

The observers sat at a distance of roughly 64 cm from the CRT display. They started a session by clicking a green button in the interface of PsychoPy. One second after clicking the button, the first trial started. The stimulus clip was presented for 2 s. After observing the stimuli, the observers were asked to report whether the stimulus clip contained a transparent liquid or not in a two-alternative forced choice manner. The observers reported their judgment by pressing one of the assigned keys. One second after pressing the key, the next trial started. Each observer participated in two sessions with each consisting of 20 Flow Speeds × 5 Backgrounds. It took 30 min for each observer to complete the two sessions. The order of trials was pseudorandomized across the observers.

### Results and Discussion

#### Psychophysical experiment

**Figure 2.**
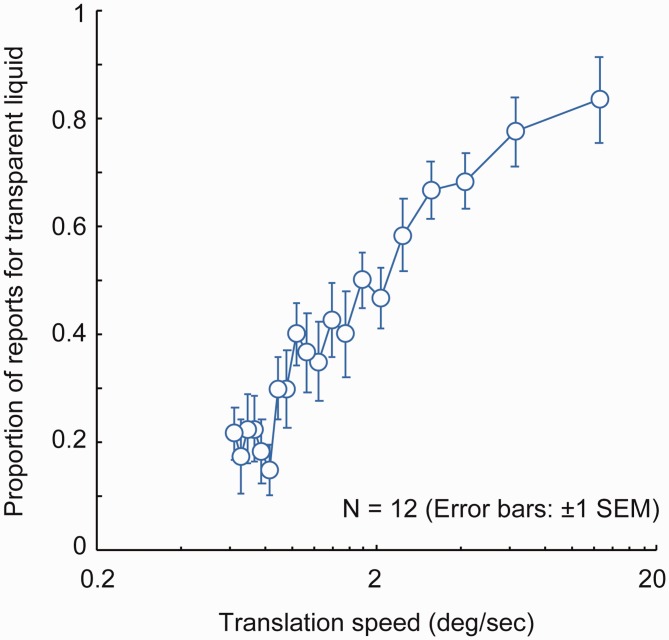
Experimental results. The proportion of reporting a transparent liquid is plotted as a function of translation speed. Error bars denote ± 1 standard error of mean (*N* = 12).

For each observer, we calculated the proportion of reporting a transparent liquid for each translation speed. Figure 2 shows the mean proportions. Using the proportions, we conducted a one-way repeated measures analysis of variance and found the significant main effect, *F*(19, 209) = 19.798, *p* < .0001. We conducted multiple comparison tests (Ryan, 1959) and found that the proportion of reporting a transparent liquid occurred more often at the higher flow speeds than at the lower flow speeds (Table 1).

**Table 1.** Significant Pairs in the Multiple Comparison Tests.

	1	2	3	4	5	6	7	8	9	10	11	12	13	14	15	16	17	18	19	20
1					*	*	*	*	*	*	*	*	*	*	*	*	*	*	*	*
2							*	*	*	*	*	*	*	*	*	*	*	*	*	*
3						*		*	*	*	*	*	*	*	*	*	*	*	*	*
4								*	*	*	*	*	*	*	*	*	*	*	*	*
5										*			*	*	*	*	*	*	*	*
6															*	*	*	*	*	*
7															*	*	*	*	*	*
8															*					
9															*	*			*	
10																				
11																				
12															*					
13																				
14																				
15																				
16																				
17																				
18																				
19																				
20																				

*Note.* Labels ranging from 1 to 20 denote the flow speeds of 0.0104°, 0.0109°, 0.0115°, 0.0122°, 0.0130°, 0.0139°, 0.0149°, 0.0160°, 0.0173°, 0.0189°, 0.0208°, 0.0231°, 0.0260°, 0.0297°, 0.03467°, 0.04160°, 0.0520°, 0.0693°, 0.1040°, and 0.2080°, respectively.

Asterisks denote the significant pairs (*p* < .05).

As expected, the proportion of those reporting a transparent liquid was higher as the flow speed increased. The results indicate that a paucity of linear motion signals in dynamic image deformations is a key factor in determining whether dynamic image deformations are perceived as a transparent liquid or not. To objectively support this hypothesis, in the next section, we calculate linear motion coverages of dynamic image deformations and assess the relationship between the proportion of reporting a transparent liquid and the linear motion coverage.

#### Analysis of linear motion coverages

**Figure 3.**
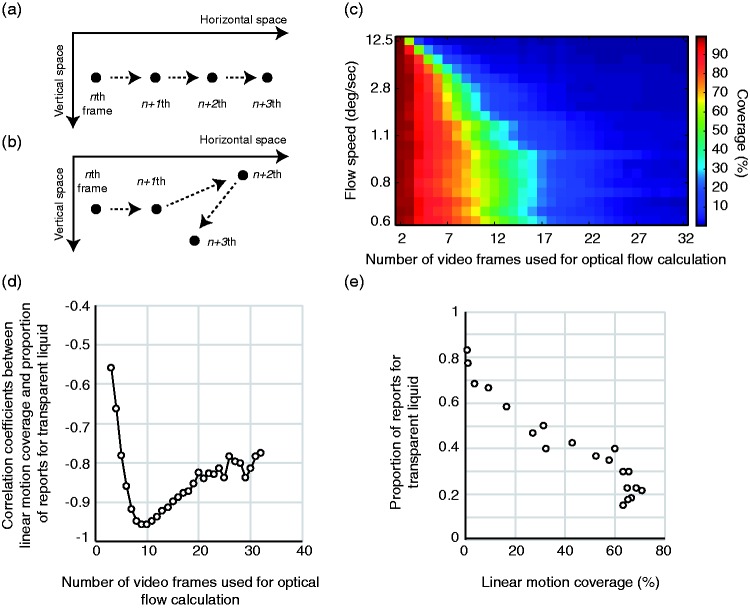
(a) Object trajectory with linear motion signals. (b) Object trajectory without linear motion signals. (c) Linear motion coverages as functions of the flow speed of image deformations (i.e., vertical axis) and the number of video frames used for optical flow calculation (i.e., horizontal axis). (d) Correlation coefficients between linear motion coverages and the proportion of reporting a transparent liquid and (e) Correlational plot between the proportion of reporting a transparent liquid and linear motion coverages when nine frames were used for the calculation.

As shown in Figure 3, i the present study, linear motion refers to motion signals with a consistent motion direction. In contrast, Figure 3(b) shows an object motion trajectory without a consistent motion direction. We focused on linear motion coverages which refer to the proportion of linear motion vectors in optical flow fields. To calculate linear motion coverages, we used the algorithm proposed by Gautama and Van Hulle (2002), which has been used in a psychophysical study to show the role of linear or nonlinear motion signals in estimating surface matte or glossiness (Doerschner et al., 2011).

Figure 3(c) shows the linear motion coverages as functions of the flow speed of image deformation and the number of video frames used for the calculation of optical flow fields. On average, the linear motion coverages increased as the flow speed decreased. The results are consistent with our prediction. Moreover, the linear motion coverages decreased with the number of video frames used for the calculation; this was because dynamic image deformations in our stimuli tended to produce more nonlinear motion signals as the stimulus duration increased.

To check how the relationship between the proportion of reporting a transparent liquid and the linear motion coverages varied with the number of video frames used for calculation, we computed their correlation coefficients (Figure 3(d)). The correlation coefficients were basically negative, and at all numbers of video frames, were statistically significant (at least *p* < .01). Above all, the correlation coefficients peaked when the number of video frames was 10 (*r* = −0.957, *t*(18) = −13.978, *p* < 4.17262 × 10^−11^). In Figure 3(e), we plotted the proportion of reporting a transparent liquid and the linear motion coverages when the number of video frames for calculation was 10.

The results clearly showed that the proportion of reporting a transparent liquid was negatively correlated with the linear motion coverages of dynamic image deformations, suggesting that human observers use linear motion coverages as a cue to judging whether dynamic image deformations originate from a transparent liquid or not. Moreover, the negative correlation peaked when the number of video frames used for calculation was around 10. In this study, the duration of a single video frame was 16.7 ms. Therefore, the results indicate that the visual system recruits motion signals across approximately 167 ms to judge the source of dynamic image deformations. Because as shown in Figure 3(d), the function of the correlation coefficients was inversely heavily tailed along the axis of the number of video frames, the visual system may use motion signals over longer durations to make a judgment about it.

## General Discussion

Image motion is one of the strong cues the visual system uses to judge material properties (Bi, Jin, Nienborg, & Xiao, 2018; Bi & Xiao, 2016; Doerschner et al., 2011; Kawabe et al., 2015; Morgenstern & Kersten, 2017). The present study also proposed that the coverage of linear motion signals is used by the visual system to judge whether dynamic image deformations come from a transparent liquid or not.

Why linear motion coverages decreased with the number of video frames used for calculation is a topic worthy of close discussion. When the deformation vector maps flowed horizontally, the texture of background images was heterogenous along the flow trajectory. Due to the background heterogeneity in terms of orientation and spatial frequency, motion directions along the flow trajectory likely changed across frames. This variation in motion directions was possibly the source of nonlinear motion. As such, it is expected that the spatial characteristics of background images such as spatial frequency or texture density would easily change the critical parameters of dynamic image deformations to cause the perception of a transparent liquid. The relationship between the spatial frequency of background images and the spatial frequency of dynamic image deformation may also alter the critical parameters. Future studies are necessary to solve these issues.

We speculate that the visual system extracts multiple information from dynamic image deformations to ascertain various attributes of transparent materials. As previous studies have already reported, human observers can perceptually discriminate liquid from hot air on the basis of the magnitude of image deformation (Kawabe & Kogovsek, 2017). In other words, the visual system seems to exploit the magnitude of image deformations to discriminate whether a transparent material is a liquid or a gas. In addition, the spatiotemporal frequency of image deformation is a key attribute for perceptual transparency (Kawabe et al., 2015). As the present study showed, the linear motion coverages are a critical cue to the perception of a transparent liquid. Based on these findings, it is possible that the visual system simultaneously extracts multiple information from dynamic image deformation(s) and determines the various attributes of materials such as optical properties, mechanical properties, and the type of material.

We would like to mention the relationship between the perception of image deformations and the perception of a transparent liquid. When the flow of image deformation was slow, observers rarely reported seeing a transparent liquid. On the other hand, it is possible to perceive dynamic image deformations even with a clip having the slowest flow speed (Video 1). Thus, it can reasonably be assumed that the mechanism for the perception of a transparent liquid goes beyond the simple detection of motion signals to support nonrigid structures as previous studies have reported (Nakayama & Silverman, 1988a, 1988b; Weiss & Adelson, 2000). The visual system may heuristically determine material types from dynamic image deformations by relying on the statistical aspect of linear (or nonlinear) motion signals. Otherwise, the visual system may focus on the appearance of dynamic image deformations to judge the underlying source of dynamic image deformations. Further clarification is necessary to elucidate the precise mechanism involved in the perception of a transparent liquid from dynamic image deformations.
